# Resource Selection and Its Implications for Wide-Ranging Mammals of the Brazilian Cerrado

**DOI:** 10.1371/journal.pone.0028939

**Published:** 2011-12-20

**Authors:** Carly Vynne, Jonah L. Keim, Ricardo B. Machado, Jader Marinho-Filho, Leandro Silveira, Martha J. Groom, Samuel K. Wasser

**Affiliations:** 1 Department of Biology, University of Washington, Seattle, Washington, United States of America; 2 Science and Evaluation, National Fish and Wildlife Foundation, Washington, District of Columbia, United States of America; 3 Matrix Solutions Inc., Edmonton, Alberta, Canada; 4 Zoology Department, University of Brasília, Brasilia, Distrito Federal, Brazil; 5 Jaguar Conservation Fund, Mineiros, Goiás, Brazil; 6 Interdisciplinary Arts & Sciences, University of Washington Bothell, Bothell, Washington, United States of America; University of Alberta, Canada

## Abstract

Conserving animals beyond protected areas is critical because even the largest reserves may be too small to maintain viable populations for many wide-ranging species. Identification of landscape features that will promote persistence of a diverse array of species is a high priority, particularly, for protected areas that reside in regions of otherwise extensive habitat loss. This is the case for Emas National Park, a small but important protected area located in the Brazilian Cerrado, the world's most biologically diverse savanna. Emas Park is a large-mammal global conservation priority area but is too small to protect wide-ranging mammals for the long-term and conserving these populations will depend on the landscape surrounding the park. We employed novel, noninvasive methods to determine the relative importance of resources found within the park, as well as identify landscape features that promote persistence of wide-ranging mammals outside reserve borders. We used scat detection dogs to survey for five large mammals of conservation concern: giant armadillo (*Priodontes maximus*), giant anteater (*Myrmecophaga tridactyla*), maned wolf (*Chrysocyon brachyurus*), jaguar (*Panthera onca*), and puma (*Puma concolor*). We estimated resource selection probability functions for each species from 1,572 scat locations and 434 giant armadillo burrow locations. Results indicate that giant armadillos and jaguars are highly selective of natural habitats, which makes both species sensitive to landscape change from agricultural development. Due to the high amount of such development outside of the Emas Park boundary, the park provides rare resource conditions that are particularly important for these two species. We also reveal that both woodland and forest vegetation remnants enable use of the agricultural landscape as a whole for maned wolves, pumas, and giant anteaters. We identify those features and their landscape compositions that should be prioritized for conservation, arguing that a multi-faceted approach is required to protect these species.

## Introduction

Emas National Park is one of the most important protected areas in the Brazilian Cerrado and the greater Park landscape is a global priority for large-mammal conservation because it is one of only 12 places in all of South America that has an intact large mammalian fauna [1]. While the Park, at 1320 km^2^, is by itself too small to protect populations of large mammals [2], [3], Brazilian federal law requires landowners to leave 20% of their farm's original vegetation intact [4]. This system of private lands under conservation may be responsible for the continued presence of the landscape's wide-ranging mammals, yet it is unknown whether their continued presence in this region can be credited to adequate habitat protection. Indeed, some species may have stable populations in the region while others may be in decline owing to species-specific differences in landscape requirements. Understanding the role of the private lands in conserving species is urgent since there is a pressure from the Brazilian agribusiness sector to weaken the federally-mandated private lands conservation scheme (the Forest Code), which currently requires landowners in this region to leave at least 20% of the native vegetation intact [5]. A priority of our research was to understand if the existing system of conserved habitat on private lands was enabling resource use by the study species.

In a broader context, large, wide-ranging mammals often play disproportionately large roles in their ecosystems making their conservation of particular concern [6]–[9]. They are prone to local extirpation [10], [11] and 39% of large mammals (body mass >20 kg) are considered threatened with extinction [1], compared with 25% for mammals as a whole. Conservation planning at the landscape scale is crucial to the persistence of wide-ranging mammals because their populations can rarely be conserved for the long-term solely in protected areas [11]. Yet, achieving conservation goals across multiple locales, scales, and landowners is often complicated.

To improve understanding of the contribution of private and protected land management to large-mammal conservation in the Cerrado, we analyzed resource selection patterns for five wide ranging mammals of conservation concern: giant armadillo (*Priodontes maximus*), giant anteater (*Myrmecophaga tridactyla*), maned wolf (*Chrysocyon brachyurus*), jaguar (*Panthera onca*), and puma (*Puma concolor*). We used detection dogs trained to simultaneously sample for scat from the five study species inside and outside Emas National Park, taking advantage of the dogs' exceptional ability to locate scat samples from multiple species over large remote areas [12]–[14]. Under the assumption that these species select resources for fitness advantages, resource selection probability functions [15] were estimated from sample locations and covariates of landscape composition to determine the types of conditions that would promote their conservation in the Cerrado. Our study represents the first study on resource selection for all five of these species in the Cerrado, where extensive and ongoing land conversion has already resulted in a precarious future for the study species [16].

### Study area and species

The Brazilian Cerrado comprises 21% of Brazil and is the world's largest, richest, and most threatened tropical savanna [17]. More than 50% of its approximately 2 million km^2^ has been directly cleared for large-scale agriculture and livestock grazing in the past 40 years [4] and this conversion now represents the world's single largest increase in farmland since the early 1900s settlement of the U.S. Midwest [18]. Both the total amount and annual rate of clearing are higher here than in the Brazilian Amazon and only 2.2% is legally protected in parks and reserves [4].

**Figure 1:**
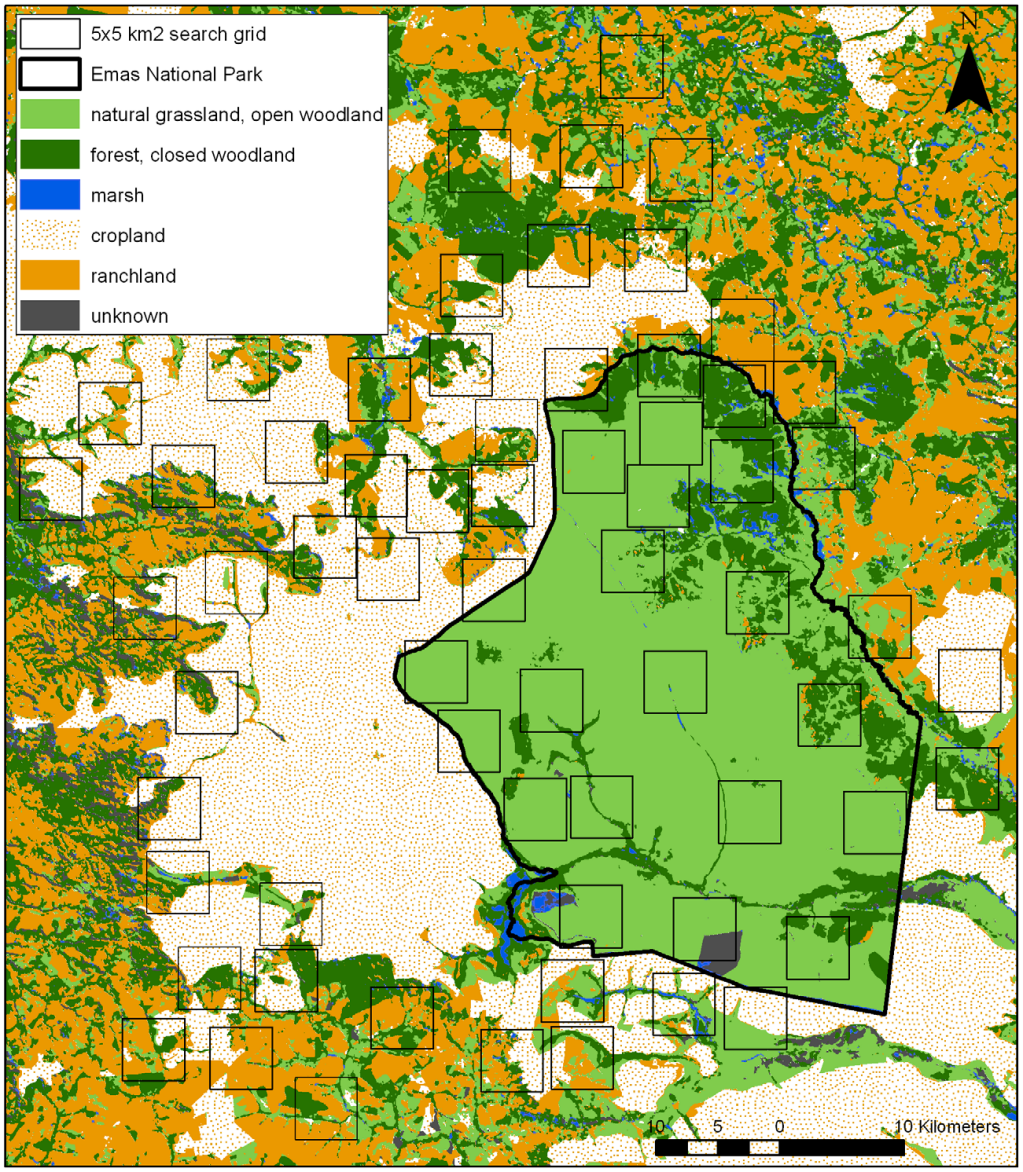
Survey quadrats and habitat types occurring in and around Emas National Park, Brazil.

Our study area spans 4600 km^2^ of private farmland (predominately soy, corn, and cotton), cattle pasture, and Emas National Park, a federally-protected reserve in the tri-state region of Goiás, Mato Grosso, and Mato Grosso do Sul States (18°19′S, 52°45′W), Brazil. Grasslands were once the dominant vegetation type in the natural mosaic of savanna and woodlands that comprises the study area. However, they have been nearly entirely replaced by large-scale agriculture and cattle pastures on private lands (Fig. 1). Croplands are typically monocultures, planted and harvested biannually, and farms in the study area are on average about 30 km^2^ with some as large as 200 km^2^. Remnant vegetation on private farms includes forested riparian corridors that buffer the region's extensive river system, seasonally inundated grasslands (marsh), and patches of woodland fragments (Fig. 1). Rainfall in the region is extremely seasonal, with most of the 1500 mm of annual rainfall occurring between the months of October and March [18].

Our study area also includes the entire Emas National Park, a 1320 km^2^ IUCN category II protected area that conserves large tracts of grassland plains and open shrublands (81%), woodlands and riparian forest (17%), and marshlands (1%; Fig. 1). The park is considered one of the most important protected areas in South America for large mammal conservation [19]. Wildfires sweep through the park every three or four years, burning the majority of the grasslands at regular intervals [20]. The park contains some of the largest expanses of grassland plains in the entire Cerrado biome, and is thus particularly important for grassland-endemic and obligate species [21].

Each of our study's five focal species is considered ‘at-risk’ in Brazil [22], and all except puma are considered either ‘near-threatened’ (jaguar, giant anteater, maned wolf) or ‘threatened’ (giant armadillo) by the IUCN Redlist [23]. Population trends for four of the species are decreasing and the maned wolf's population trend is unknown [23]. All five species are listed as ‘prohibited’ for international trade by the Convention on International Trade in Endangered Species under Appendix I or II [24]. Shared threats to this group include habitat conversion and isolation of populations, death due to motor vehicle collisions, disease and persecution by domestic dogs, bush meat hunting (giant armadillo), and poaching of their wild prey base (puma and jaguar; [23]).

The giant armadillo, Family Dasypodidae, is the largest armadillo species, weighing an average of about 35 kg [25]. Their home range in this region is estimated at 10 km^2^ and local minimum density is 3.36 individuals/100 km^2^ [26]. Like the giant armadillos, giant anteaters, Family Myrmecophagidae, are highly adapted for feeding on ants and termites [25]. Giant anteaters weigh about 32 kg [25] and their density is estimated at 0.2–0.4 individuals/km^2^ in the park's grassland plains [27]. Due to high concentrations of their prey biomass in the grasslands, Emas National Park is thought to support some of the highest known densities of both giant armadillos and giant anteaters. Information on their habitat preferences and their distribution outside of protected areas, however, is lacking. Giant anteaters suffer mortality on roads and highways and, while this was one of the first studies on giant armadillos, they are generally considered to be sensitive to human disturbance [23].

Standing at about 90 cm at the shoulder and weighing 24 kg, the maned wolf, Family Canidae, is the largest canid is South America, where it is restricted to the central grasslands. Maned wolves are omnivores, consuming small vertebrates, invertebrates, and large quantities of fruit [28]. In Emas National Park, the density is about 5 individuals/100 km^2^ and the average home range size is about 80 km^2^ [3], [29]. Maned wolves are adapted for foraging in tall grasslands and prefer these areas to closed-canopy vegetation types [26], [30]. They are thought to be threatened by the near entire conversion of their global distribution to agriculture, yet it is unknown how agricultural expansion is affecting their population trend. Understanding whether maned wolves are habituating to agricultural landscapes is considered of utmost importance for their conservation [31], particularly since existing private land conservation schemes tend to protect vegetation types not typically used by maned wolves (mainly woodland and forest since these are the least desirable to farm).

In contrast to maned wolves, pumas and jaguars (Family Felidae) need cover for stalking their prey. While pumas are less than half the weight of jaguars, which weigh between 60 and 120 kg, there is often considerable overlap in their prey base. Home range estimates for this area are 32 km^2^ for pumas and 140 km^2^ for jaguars, with a density of 2 individuals/100 km^2^ [32]. Neither felid species has previously been studied outside of the park; both are considered threatened by isolation. A priority of our research was to understand if puma and jaguar were dependent on the protection provided by the park as a management unit, as well as if the availability of woodland and forest habitat is adequate to support these species.

## Methods

### Field surveys and species assignments

Project licensing for fieldwork in Brazil was provided by The Brazilian Institute on Environment and Natural Resources (IBAMA No. 02001.0002.15/2007-21). One to three scat detection dog teams (comprised of a dog, dog-handler, and, often, a field assistant) conducted surveys between August of 2004 and April of 2008. Our intent was to examine the influence of landscape and human disturbance features on species-specific resource use, independent of seasonality. We thus sampled and pooled data across all months and seasons. We also pooled data across years since landscape use was consistent in our study area during this time; we did not detect any major differences between years.

Sampling sites included 65, 5×5 km search quadrats (Fig. 1), which were visited by different teams throughout each sampling season. A total of 415 transects were made within the quadrats (such that most quadrats were surveyed between 6 and 7 times) and the average transect, or daily distance walked, per team was 7.6 km. The quadrats were predetermined to distribute surveys across the study area, ensuring that sampling would maximize the number of different vegetation types and land uses represented without biasing towards any particular microsites. Since additional quadrats placed in the large expanse to the west of the park were unable to be safely surveyed, we conducted some cropland surveys outside of the pre-selected areas. The actual survey path for each of the 415 transects was recorded with handheld GPS units set to mark a waypoint every 30 m and the total distance walked was 3170 km.

Dogs were trained and worked in the field according to methods described in Wasser et al. [12]. Teams walked freely (i.e., did not follow grid lines) and dogs searched off-leash within designated search quadrats. This allowed the handler to guide the dog according to wind direction and to follow a dog pursuing a scent [14]. Each dog was trained to find the scats of all target species and detection rates were consistent across teams, years, and seasons [14]. When a scat was located, the handler rewarded the dog, recorded the GPS position and data on the site and sample, and collected the sample. Scats that were <5 m apart and of the same contents and age were recorded as one sample.

GPS locations for 434 giant armadillo burrows were also recorded. Burrows are highly indicative of habitat preferences for giant armadillos because they indicate areas preferred for foraging, shelter, and protection [33]. Due to their large size, giant armadillo burrows are easily identifiable and we had previously shown that detection of burrows was not biased by habitat type [26]. The nocturnal giant armadillos use burrows nightly, show repeat use of burrows only on occasion [26], [33], and all individuals dig multiple burrows within their home range [25].

Scat samples of putative maned wolf, puma, and jaguar origin were subjected to DNA analysis for species identification since these samples could be confused either amongst each other or with other sympatric carnivores, such as ocelot (*Leopardus pardalis*) and fox (*Cerdocyon thous*). Samples were processed in the laboratory of the Center for Conservation Biology at the University of Washington (Seattle, USA) according to methods described in Vynne et al. [34]. We included in our analyses all samples that were confirmed by DNA (*n* = 952, 80, and 36 for maned wolf, puma, and jaguar, respectively). Giant anteater scats are genuinely unambiguous in terms of species identification due to their unique shape, large size, and/or contents [35]. We thus did not subject these samples (*n* = 504) to genetic analysis for species confirmation.

### Resource selection design and analysis

We employed a “use and available” study design [15], [36], [37] to estimate resource selection.

‘Used’ sites were defined by the locations where scat and giant armadillo burrows were detected and ‘available’ sites were defined by 20 000 random locations within 30 m of the sampled transects. Hence, both the used and available sites were equally constrained to within the sampled landscape. Such constrained analyses are common to resource selection studies [38].

Scat deposition by carnivores, and maned wolves in particular, may be used as territorial markings and placed within a short distance of evidence of conspecifics, such as near the edges of a territory [39], [40]. Selection of sites for scat deposition by carnivores may thus not necessarily scale with general habitat use and is unlikely to be highly indicative of microhabitat use. However, we believe that scat locations are an appropriate method for addressing broad-scale questions about the resource selection patterns of these species with respect to broad-scale, landscape covariates included in this study. Sampling with detection dogs across dispersed quadrats maximizes the number of individuals captured and these methods have been shown to accurately reflect resource use patterns when compared with studies of radio-collared individuals [12], [41]. Unlike carnivores, giant anteaters appear to defecate randomly as they forage and move about their home range (Vynne, personal observation). Thus, we assume that used locations based on giant anteater scat are indicative of overall resource use.

We analyzed both used and available sites with respect to environmental variables that we suspected might influence resource selection by one or more of the target species. Because we were interested in identifying broad-scale associations and the influence of human development on land use by these species, we assigned vegetation types to broad habitat classifications and then combined these classes to examine the influence of natural vegetation (all natural, open-canopy, closed canopy) versus human development (cropland, pasture) (Table 1). Measurement error from unclassifiable vegetation categories due to cloud cover, steep slopes, or recently burned areas comprises less than 5% of our study area and vegetation classifications are as assigned in Ferreira et al. 2003 [42].

**Table 1:** Definitions and labels for covariates tested in resource selection models.

Covariate	Label	Definition
Park	park	discrete variable; sample found inside Emas National Park
Distance to park	parkDist	distance, in meters, to the National Park
Natural water spring	spring	discrete variable; site is within 500 m of a natural water spring
Rivers	river	distance, in meters, to the nearest permanent river-like waterway
Any road	road	within 30 meters of any road, paved or unpaved
Distance to road	roadDist	distance, in kilometers, to any road, paved or unpaved
Distance to main road	MainroadDist	distance, in kilometers, to any paved or busy road
Riverine forest	forest	discrete variable; high, tall-canopy forest habitat determined by year-round high soil moisture.
Woodland cerrado	cerrado	discrete variable; closed woodland with crown cover of 50% to 90%, made up of trees, often 8–12 m or even taller, casting a considerable shade so that the ground layer is much reduced
Open cerrado	open cerrado	discrete variable; vegetation is dominated (at least visually) by trees and shrubs often 3–8 m tall and giving more than 30% crown cover but with still a fair amount of herbaceous vegetation
Open grassland	grassland	discrete variable; dry grassland without shrubs or trees or with a scattering of shrubs and small trees
Inundated marshland	marsh	discrete variable; seasonally waterlogged grasslands
Cattle pasture	pasture	discrete variable; pasture area used for grazing livestock, predominantly cattle
Agriculture	agriculture	discrete variable; agricultural land used for growing soy, corn, millet, cotton, or sugar cane
Unknown	unknown	habitat of unknown type; classification could not be determined due to cloud cover over satellite image
Distance to agriculture	agDist	distance, in kilometers, to any agricultural field
Distance to closed	closedDist	distance, in kilometers, to either forest or cerrado
Distance to edge	edgeDist	distance, in kilometers, to any habitat edge
Closed-canopy	closed	proportion of closed-canopy habitat (cerrado, forest) within 1.4 km^2^ of a sample
Open-canopy	open	proportion of non-agriculture, open-canopy habitat (grassland, open cerrado, ranchland) within 1.4 km^2^ of a sample
Natural habitat	natural	proportion of natural habitat (forest, cerrado, open cerrado, grassland, marsh) within 1.4 km^2^ of sample
Non-cropland	nocrop	proportion of non-cropland habitat within 1 km^2^ of a sample
Elevation	elevation	elevation, in meters, as analyzed from a Digital Elevation Model
Habitat heterogeneity	heterogeneous	number of different vegetation types within small, medium, and large window around sample

Multi-model inference was conducted as part of the model selection process recognizing that different model forms should be considered to determine which model best ‘fits’ the data [43]. We considered two competing model forms in estimating resource selection: the exponential resource selection function and the logistic form of the resource selection probability function (RSPF) [15], [44]. Models were estimated using maximum likelihood methods [16]. The final model form and covariates were selected in two steps. First, models selection was conducted using Bayesian Information Criterion (BIC) since we were interested in both the relative importance of variables and model prediction [45]–[49]. Second, the distribution and range of the predicted values was explored to confirm that the selected model, based on BIC, did not contain anomalies (e.g. such as maximum probabilities of selection near zero or a confined distribution of probability values). The final model determines the model form and set of covariates that best estimates resource selection for each study species.

We use boxplots to show how each species selects resources by plotting the predicted values of the final resource selection models in relation to habitat classifications, the proportion of available natural habitat within 1.4 km of a sample, the proportion of available forested habitat within 1.4 km of sample, and the presence of the national park. These plots show the middle two quartiles of the data, the median, represented by the line within the box, and the 90th and 10th percentiles of the range, represented by error bars.

## Results

All of the final models are in the form of the logistic resource selection probability function wherein the function gives the probability that a particular resource unit, as characterized by a combination of environmental variables, will be selected by an individual animal given that it is encountered [15]. Table S1 provides the BIC differences for the various models considered in selecting the final model for each species. Table 2 provides the parameter estimates and standard errors for the final models for each of the species. The final models are consistent with other studies of habitat use by the target species [26], [28], [30], [32], [50]. This consistency supports our assumption that we can identify broad scale landscape distribution patterns from scat (for giant anteater, maned wolf, puma and jaguar) and giant armadillo burrow locations.

**Table 2:** Parameter estimates and standard errors for the final resource selection models for species surveyed in the Cerrado of Brazil.

Species	Covariate	Parameter Estimate	Standard Error
Armadillo	intercept	−2.232	0.425
	closedDist	0.514	0.220
	natural	0.747	0.619
	marsh	−3.910	1.668
	MainroadDist	−0.729	0.191
	natural * MainroadDist	1.894	0.375
Anteater	intercept	−1.842	0.380
	nocrop	−0.931	0.569
	roadDist	−0.086	0.078
	forest	1.287	0.456
	cerrado	0.779	0.432
	open cerrado	1.858	0.480
	pasture	0.943	0.250
	unknown	1.883	0.997
	nocrop*roadDist	0.806	0.236
Maned wolf	intercept	2.391	0.573
	agDist	−1.267	0.184
	(agDist)^2^	0.122	0.019
	closed	−2.913	0.524
	pasture	−1.176	0.311
Jaguar	intercept	−8.387	2.100
	closed	2.670	1.830
	natural	7.469	2.398
Puma	intercept	−4.084	1.208
	closed	25.620	8.307
	natural	2.508	1.371

Positive parameter estimates indicate a positive relationship between the covariate and resource selection. All of the final models are in the form of the logistic resource selection probability function.

### Resource selection by giant armadillos

**Figure 2:**
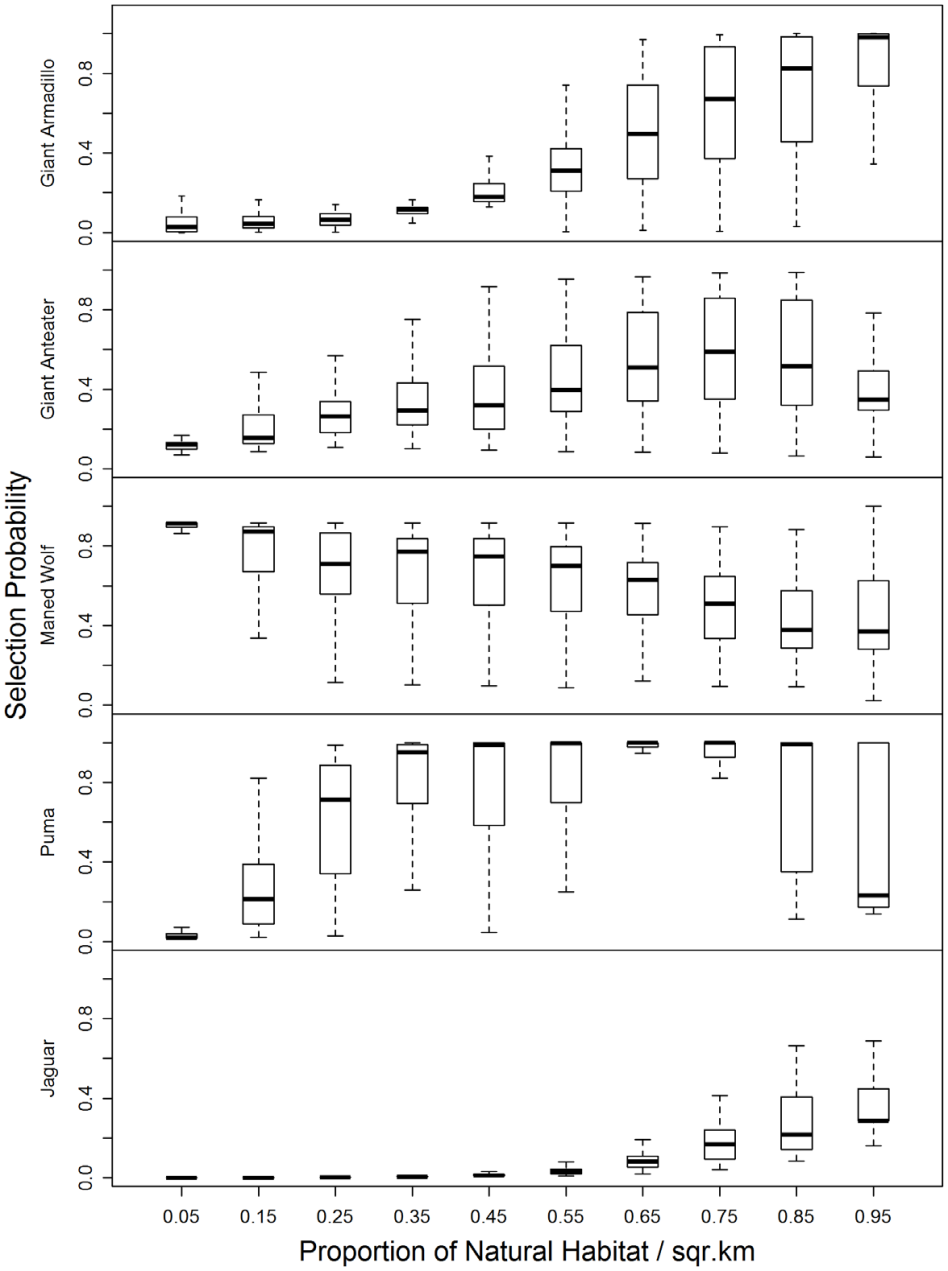
Selection probability by species and proportion of natural habitat per square kilometer.

**Figure 3:**
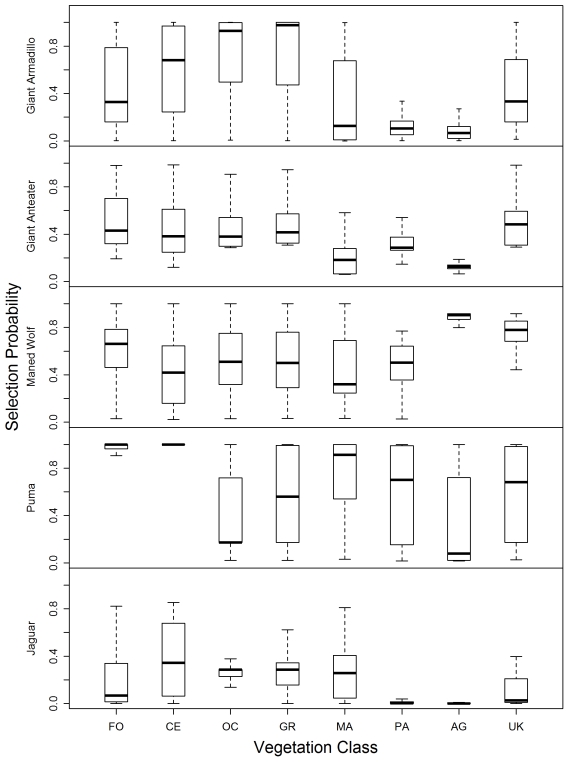
Selection probability by species and vegetation type (FO = forest, CE = cerrado, OC = open cerrado, GR = grassland, MA = marshland, PA = pasture, AG = cropland, UK = unclassified/unknown).

**Figure 4:**
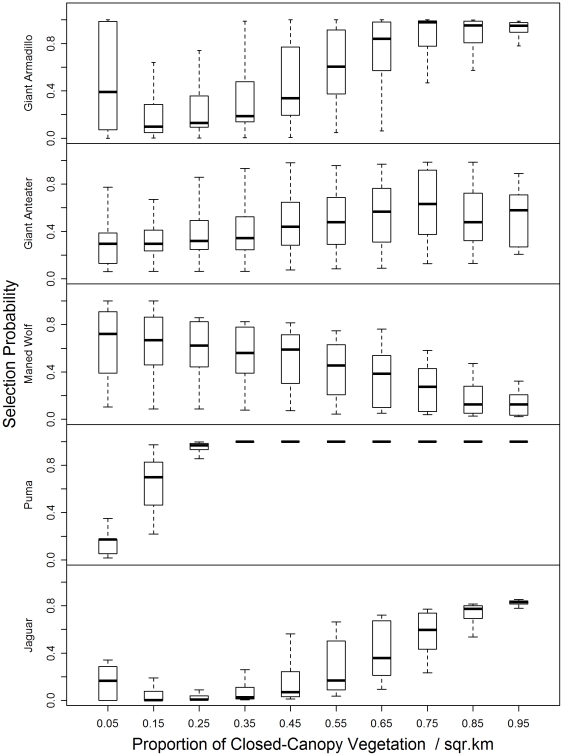
Selection probability by species and proportion of closed-canopy vegetation per square kilometer.

**Figure 5:**
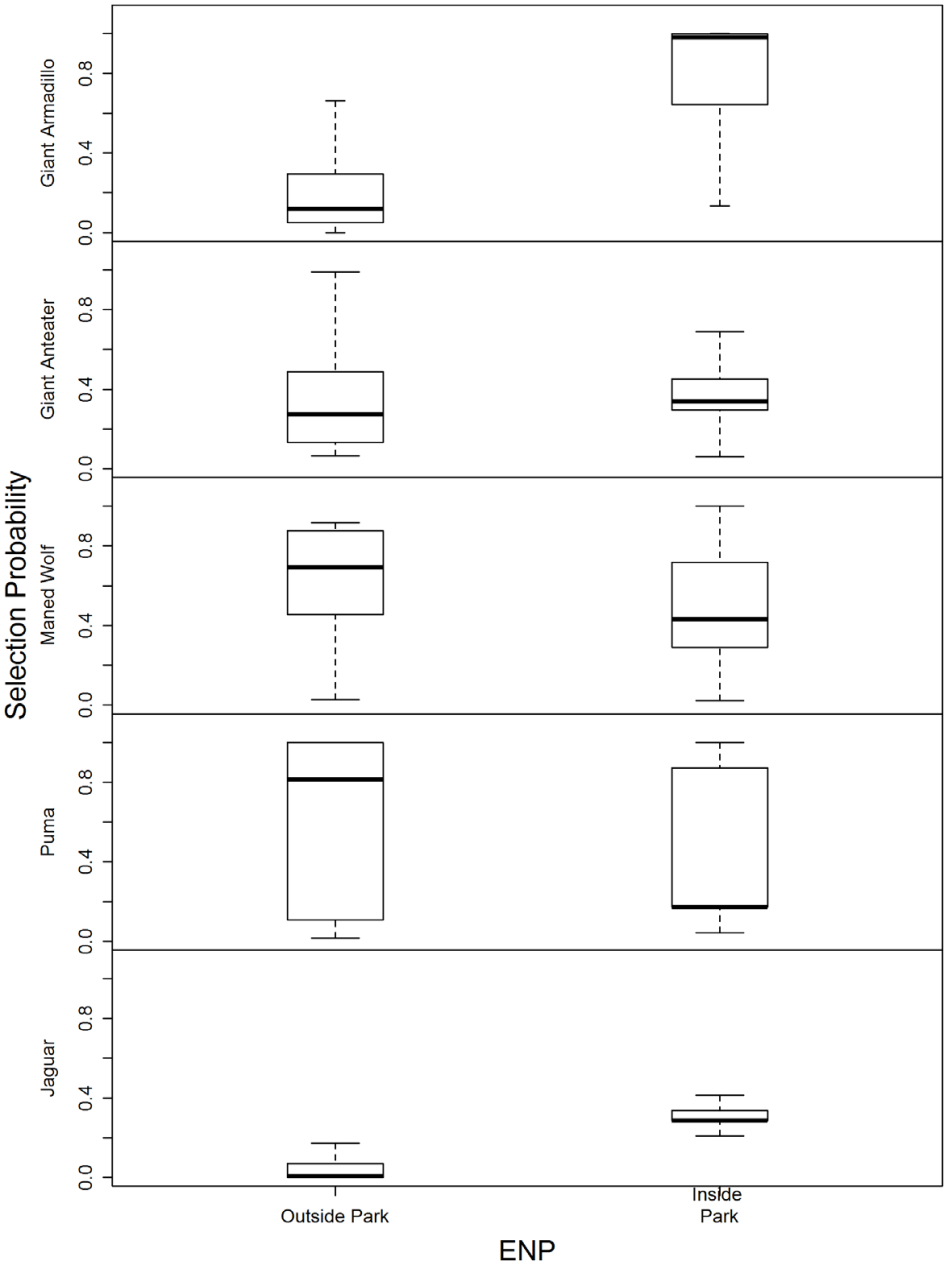
Selection probability by species and outside versus inside Emas National Park.

The resource selection model for giant armadillos revealed the importance of natural landscape conditions to the distribution of this species. Giant armadillos selected areas that were more natural and far from busy roads (Table 2; Fig. 2). Their preferred habitat types were grasslands and open woodlands, and they avoided burrowing in wet soil conditions and developed areas (marsh; Fig. 3). Giant armadillos also strongly selected areas that supported a high proportion (>60%) of intact natural forest (Fig. 4). Their presence on the landscape outside of the park, where grasslands are poorly preserved (Fig. 1, Table 1) is likely to be dependent on the presence of conserved forest tracts. Due to the giant armadillos' strong preference for natural habitats, the highly selected conditions for giant armadillos were overwhelmingly concentrated within the park (Fig. 5). Interestingly, when in non-preferred habitats, giant armadillos changed their selection patterns with respect to roads, favoring proximity to roads when in croplands (Table 2). We expect this is because roads are more stable than croplands, which are subject to bi-annual plowing.

### Resource selection by giant anteaters

Giant anteaters selected areas that were further from roads and that were more natural (Fig. 2). This relationship was non-linear and had a strong interacting effect wherein anteater selection was positively influenced by these combined relations (Table 2). As with the giant armadillo, when the habitat conditions changed to have increased cropland influence then the anteaters were more likely to select for sites nearer to roads. This is likely because they were using roads as travel corridors through croplands that provide low foraging potential. Highly selected vegetation classifications included grasslands, open cerrado, forest, and woodland cerrado (Table 2; Fig. 3). When anteaters were within their preferred grassland habitat, they used areas regardless of the proximity to forest. Vegetation types other than grassland were selected only when they were within about 1 km of forest or woodland, vegetation types that other studies have shown were important for temperature buffering [50], [51] and bathing [52]. Thus, outside of the park, the federally-protected riparian forests were a key feature whose presence enabled use of the landscape generally by giant anteaters.

**Resource selection by maned wolves:** The estimated RSPF for maned wolves was dominated by proximity to agriculture and avoidance of closed-canopy habitats (Table 2). Maned wolves selected strongly for scat deposition in farmed fields and the probability of use decreased with distance to cultivated field. This, however, was a quadratic relationship; the distance to nearest farm no longer affected selection when maned wolves were >5 km from agriculture. Since the only region in our study site >5 km from agriculture was in Emas National Park, we interpret this result to mean that maned wolves were just as likely to select habitats within the core of the park (natural grasslands) as they were to select for agriculture. Thus, while the open natural grasslands of the park were a preferred habitat type for maned wolves, when the wolves were near (<5 km) croplands they were displaced to these areas, likely for foraging benefits.

That maned wolf scat locations outside of the park tended to have a higher selection probability than those found inside the park (Fig. 5) appeared to be driven by the availability of croplands outside park borders. Maned wolves also increasingly and strongly avoided areas as the proportion of closed-canopy habitat reached ≥30% (Fig. 4). The avoidance of closed-canopy is noteworthy since the current practice of leaving predominately forests and woodlands in conservation on private lands is likely to benefit maned wolves less than would the conservation of grasslands.

### Resource selection by puma and jaguar

Jaguar scats were mainly found along rivers in the forested areas of the park, consistent with findings by Silveira [32]. Puma were found in all habitat types, though they were most commonly located in cerrado (31% of locations), open cerrado (24% of locations), and ranchland (16% of locations). The amount of natural habitat and, especially, the amount of closed-canopy habitat drove selection by both jaguars and pumas for scat deposition (Fig. 2; Fig. 4). Pumas were most strongly influenced by the amount of closed-canopy habitat available, whereas jaguars had the strongest association with natural habitat (which includes both closed-canopy and native grasslands, Table 2). Thus, while resource selection by puma was not influenced by management unit, the presence of the park appears critical for jaguar (Fig. 5). Puma resource selection was influenced by the amount of conserved forest fragments, which are likely important for them for stalking prey (Fig. 4). Also, jaguars were associated with a much higher percentage of closed-canopy vegetation types when outside the park than when inside it, suggesting that they require larger areas of woodland or forest when outside of sites managed for conservation.

## Discussion

Persistence of wide-ranging animals in highly developed landscapes often depends on their ability to use fragments of natural habitat and move across complex landscapes to find essential resources [53], [54]. Resource selection models revealed several distinct ways in which species used this changing Cerrado landscape, and allow us to draw several key inferences.

Giant armadillos and jaguars strongly avoid human disturbance and both are tied to the park as a management unit. Giant armadillos' strong association with the park is likely due to the presence of natural habitat, which is the main driver of their resource selection model. The park also provides a refuge from vehicular traffic, since the park experiences <5 vehicular trips per day. We believe that jaguars are particularly sensitive to noise and human disturbance that is prevalent in the agricultural areas that interact with all except the largest tracks of protected habitat on the landscape outside of the park [55], [56]. This may be why jaguars required larger tracts of intact woodland and forest to be present outside of the park than inside of it. The park is therefore important for jaguars since it protects the largest tracts of natural habitat and provides a refuge from disturbance associated with croplands.

Our results show that Federally-mandated set-asides on private lands are crucial for large mammal conservation in the Cerrado. Pumas use of the beyond-park landscape appears closely tied with availability of stalking cover and security since they were highly associated with edge habitats near woodlands and forests. Giant anteaters' use of the beyond-park landscape was also closely tied to proximity to forest. Behavioral observations have shown that anteaters tend to do active feeding in open areas (where food resources are concentrated) and select forested sites for resting and temperature buffering [50], [51]. This likely explains why distance to closed-canopy forest was important for giant anteaters. It may also explain why presence of remnant habitat is particularly important when giant anteaters are in non-preferred agricultural areas, since these areas are subject to the highest temperature extremes. Maned wolves, which generally avoid areas dominated by dense canopy, rest in dense vegetation during the day [30], [57] and the presence of resting areas amongst the agricultural fields is likely what has allowed this species to use the cropland-dominated landscape.

While the remnant vegetation left on private lands is critical, our results suggest that the 20% threshold may not be adequate to conserve the full suite of large mammalian fauna for perpetuity. Giant armadillo and jaguar, in particular, only select areas for use once the amount of natural habitat available is greater than 50%. Puma require at least 15% of the vegetation within a square kilometer to be closed-canopy vegetation and selection of areas by jaguar increases strongly once the amount of available closed-canopy vegetation is more than 60%.

Besides protecting remnant woodlands and forests, future conservation planning or mitigation efforts should aim to increase availability of underrepresented vegetation-types. Open grasslands were once the dominant habitat type in the region but now comprise less than 10% of the remaining habitat fragments in our study area outside the park because they are the most desirable to farm [14]. Yet giant armadillos, giant anteaters, and maned wolves all are highly associated with such vegetation-types. We urge that the Forest Code Law be extended to ensure that the private preserves be representative of the original landscape of the farm holding, thus ensuring that remaining grasslands on private farms be conserved or restored. Using government or private schemes for purchasing or paying for easements of any privately held, remaining grasslands also would help conserve the grassland-adapted species of the Cerrado.

Understanding the contribution of croplands to the persistence of maned wolves, in particular, is critically important. We suspect that individuals are attracted to the croplands for foraging on rodents: maned wolves rarely consumed the crops themselves and the diet of individuals living in the landscape outside of the park consisted nearly entirely of rodents and seeds of *Solanum lycocarpum* (Vynne, unpublished data). Based on hormone analysis, animals using croplands had high nutritional status indicating a potential benefit of maned wolf use of cropland habitats [58]. Yet, croplands are not benign habitats. Disturbance due to presence of humans and active machinery, ingestion of pesticides, direct interactions with domestic dogs, and increased exposure to disease may all combine to adversely affect survival and reproduction in the croplands. Since maned wolf population trends are virtually unknown [59] and the majority of their range overlaps with agriculture, understanding the influence of cropland use on their fitness is of utmost priority.

Follow-up monitoring in this region is necessary to ensure populations are stable and not in decline following the relatively recent conversion and use intensification of the region. This is particularly important given that the croplands are currently undergoing another major shift from low-growing soy plantations to sugar cane, which produces a denser, darker canopy. The more labor-intensive requirements of growing the sugar cane and increased human presence in the region may make the landscape mosaic more hostile as a place of residence for species that are sensitive to disturbance or hunting, particularly giant armadillo, giant anteater and jaguar. Sugar cane development is also likely to have adverse implications for maned wolves, which strongly avoid closed-canopy habitats. A recent study examining maned wolf adult survival rates over 16 years showed a decline in the adult maned wolf survival rates corresponding to years of increased sugar cane expansion [59]. Monitoring and planning for anticipated effects of sugar cane expansion on maned wolves and other species of concern would be enabled by periodic evaluations of resource selection at various intervals of land use change.

While we assumed that species select resource units for fitness advantages, the selection of habitats by wildlife does not always mean that a fitness advantage results [60], [61]. Where source-sink dynamics are present, resource selection models may predict a high probability of selection, but those locations may negatively affect population productivity. For example, maned wolves' selection of agricultural areas may provide a foraging advantage but have overall fitness costs due to increased toxicity or physiological stress. Given the amount of changing landscape use and development in this area, future studies should combine resource selection results with measures of health and fitness to better understand the consequences and mechanisms of resource selection by these species. Recent studies have demonstrated that combining resource selection studies with information on life requisites, including physiological health and fitness, may better inform conservation management by providing key insights into the mechanisms and consequences of resource selection [41], [58], [62].

In conclusion, since much of the Cerrado biome is degraded, and most reserves are too small to solely ensure the preservation of their large mammalian fauna, understanding the role of the landscape mosaic and managing private lands for conservation is critical. Our data support previous claims that if existing laws were applied efficiently, the resulting habitat fragments could support some Cerrado species [63]. Furthermore, our analyses provide some of the first data for these species in the region and show that the continued presence of this suite of large, wide-ranging mammals in the Emas Park region is likely due to a combination of a well-managed reserve and an extensive network of habitat remnants in the form of forested river corridors and patches of woodland. The varied habitat preferences of this suite of species demonstrate the multi-faceted approach that will be required to achieve comprehensive conservation outcomes.

## Supporting Information

Table S1 **BIC differences for various giant armadillo, giant anteater, maned wolf, jaguar, and puma resource selection models.**
(DOCX)
