# Supplementary material for: Resource Selection and Its Implications for Wide-Ranging Mammals of the Brazilian Cerrado
Source: PLoS One. 2011 Dec 20;6(12):e28939. doi: 10.1371/journal.pone.0028939 (PMC3243687; doi:10.1371/journal.pone.0028939)
Supplement: Table S1 — BIC differences for various giant armadillo, giant anteater, maned wolf, jaguar, and puma resource selection models. (DOCX) [file pone.0028939.s001.docx]

Table S1. BIC differences for various giant armadillo, giant anteater, maned wolf, jaguar, and puma resource selection models.

| Species |  |  | Candidate Models | Logistic RSPF | Exponential RSF |
| --- | --- | --- | --- | --- | --- |
| Armadillo |  |  | natural | -53.64 | -80.51 |
|  |  |  | agDist | -174.49 | -319.89 |
|  |  |  | MainroadDist | -209.82 | -209.82 |
|  |  |  | closed | -288.61 | -288.61 |
|  |  |  | closedDist | -308.36 | -308.36 |
|  |  |  | marsh | -326.41 | -326.41 |
|  |  |  | natural + MainroadDist | -33.50 | -40.17 |
|  |  |  | natural * MainroadDist | -1.10 | -45.62 |
|  |  |  | agDist * MainroadDist | -210.40 | -210.40 |
|  |  |  | natural * MainroadDist + closed+marsh | -3.80 | -11.78 |
|  |  |  | natural * MainroadDist + closedDist + marsh | 0.00 | -46.02 |
| Anteater |  |  | nocrop | -21.63 | -69.24 |
|  |  |  | natural (cor with nocrop = 0.99) | -26.03 | -74.14 |
|  |  |  | roadDist | -20.26 | -73.25 |
|  |  |  | MainroadDist | -72.20 | -65.95 |
|  |  |  | VegetationClass | -81.62 | -81.62 |
|  |  |  | closed | -88.31 | -88.82 |
|  |  |  | Nocrop + roadDist | -19.93 | -13.84 |
|  |  |  | Nocrop * roadDist | 62.33** | -3.40 |
|  |  |  | Nocrop * roadDist + vegetation types | 0.00 | -5.35 |
|  |  |  | Nocrop * roadDist + vegetation types + closed | -0.58 | -8.71 |
| Maned wolf |  |  | closed | -145.38 | -159.99 |
|  |  |  | agDist | -181.33 | -174.47 |
|  |  |  | Ranchland | -201.28 | -194.42 |
|  |  |  | MainroadDist | -188.45 | -181.54 |
|  |  |  | roadDist | -204.08 | -197.22 |
|  |  |  | natural | -154.64 | -153.96 |
|  |  |  | agDist + agDist^2^ | -56.94 | -86.52 |
|  |  |  | closed + agDist + agDist^2^ | -15.56 | -52.60 |
|  |  |  | closed + agDist + agDist^2^ + pasture | 0.00 | -48.46 |
|  |  |  | closed + agDist + agDist^2^ + pasture + natural | -1.97 | -52.01 |
|  |  |  | closed + agDist + agDist^2^ + pasture + MainroadDist | -6.84 | -54.64 |
| Jaguar |  |  | closed | -36.64 | -38.02 |
|  |  |  | natural | -0.37 | -2.73 |
|  |  |  | closed + natural | 0.00 | -1.03 |
| Puma |  |  | closed | -0.75 | -16.30 |
|  |  |  | natural | -21.03 | -38.38 |
|  |  |  | closed + natural | 0.00 | -20.04 |

BIC difference for each candidate model was calculated from the best-fit model for each species; best-fit model has a BIC difference of 0.00. A model with a larger BIC difference is considered to provide a better fit, based on BIC. See Table 1 for abbreviation definitions. Only one of MainroadDist and roadDist could be selected to maintain independence between covariates. Only one of closed and closedDist could be selected to maintain independence between covariates. Only one of nocrop and natural could be selected due to correlation (r=0.98). **Model was not selected as best-fit because exploration of the predicted values revealed an anomaly in the distribution of probabilities: a binary distribution of selection probabilities near 0 and near 1.
